# The Non-Paced 3-Minute Sit-to-Stand Test: Feasibility and Clinical Relevance for Pulmonary Rehabilitation Assessment

**DOI:** 10.3390/healthcare11162312

**Published:** 2023-08-16

**Authors:** Rachel Ernst, Benoit Bouteleux, Marie Malhouitre, Léo Grassion, Maéva Zysman, Pauline Henrot, Mathieu Delorme

**Affiliations:** 1 Resp’Air, 33400 Talence, France; 2 Département de Pneumologie, Hôpital du Haut-Lévêque, CHU de Bordeaux, 33604 Pessac, France; 3 Centre de Recherche Cardio-Thoracique de Bordeaux, Université de Bordeaux, INSERM U1045, CIC1401, 33604 Pessac, France; 4 Service des Explorations Fonctionnelles Respiratoires, Département de Physiologie, CHU de Bordeaux, 33604 Pessac, France; 5 Direction des Actions Médicales, AFM-Téléthon, 91000 Evry, France

**Keywords:** sit-to-stand test, health-related quality-of-life, chronic obstructive pulmonary disease, exercise training, pulmonary rehabilitation

## Abstract

Pulmonary rehabilitation (PR) improves health-related quality-of-life (HRQoL) in individuals with chronic obstructive pulmonary disease (COPD), notably by increasing exercise tolerance. Easy-to-implement sit-to-stand tests can facilitate the assessment of exercise tolerance in routine practice. This retrospective study conducted in a real-life setting was designed to describe the non-paced 3-min sit-to-stand test (3-STST) and to evaluate its relationship with HRQoL (VQ11 questionnaire) to identify the determinants of 3-STST performance and to analyze the evolution of 3-STST performance and HRQoL over the course of a community-based PR program. Seventy-one COPD patients (age 69 ± 10 years old; 51% with GOLD spirometric stages III–IV) were included. Mean ± SD 3-STST performance at the initial PR assessment was 43 ± 15 repetitions. This performance was significantly associated with HRQoL and other indicators of clinical severity (lung function, dyspnea, and functional capacities). During the multivariate analysis, younger age, exertional dyspnea with mMRC ≤ 1, and better HRQoL were significantly associated with better 3-STST performance. From the initial to second PR assessment, changes in 3-STST performance were significantly associated with changes in HRQoL. This study provides evidence that the non-paced 3-STST is feasible and might be clinically relevant in the assessment of patients with COPD referred for community-based PR. This test deserves to be prospectively validated.

## 1. Introduction

Pulmonary rehabilitation (PR) improves health-related quality-of-life (HRQoL) in individuals with chronic obstructive pulmonary disease (COPD) [1]. Exercise training is a core component of any PR program and should include both aerobic (i.e., walking and/or cycling) and resistance (strength) training [2]. In order to determine the intensity of exercise training at the individual’s level as well as the objective benefits derived from a PR program, valid, reliable, and responsive assessment tools are necessary. Cardiopulmonary exercise testing and isokinetic assessment of quadricipital strength represent the gold standards for evaluating a patient’s aerobic and resistance capacities, respectively [3,4]. Nevertheless, such assessment tools require expensive and sophisticated equipment, thus restricting their accessibility and/or delaying the evaluation time before initiating a PR program.

At a time when access to PR represents a major challenge in the management of individuals with COPD [5,6], home- or community-based interventions requiring minimal equipment may provide valuable alternatives to in-patient PR programs [7,8]. The 6-min walking test (6-MWT) is currently the most validated and widely used field test for evaluating exercise capacity in patients undergoing PR, but its application remains complex [9]. Simple field tests, being easy to implement in routine clinical practice, are therefore highly desirable. To meet this expectation, several chair rise tests have been described such as the five-repetition sit-to-stand test (5-STST) [10], the 1-min sit-to-stand test (1-STST) [11], and the paced 3-min sit-to-stand test (paced 3-STST3-CRT) [12]. The latter, described by Aguilaniu et al., consists of standing up and sitting down from a chair as many times as possible over 3 min, with the pace during the first minute being imposed [12]. The pacing of the first minute (12, 15, or 20 rises) is intended to reduce variability due to patient comportment at the beginning of the test [12]. However, this implies anticipating the pace that will be imposed on the patient according to the expected physical capabilities [13,14]. It has been suggested that the choice of the pace could be based on the clinical severity of the patients, using lung function as a guide, but with different thresholds from one study to another [13,14]. Moreover, imposing a pace for the first minute may prevent comparison between groups of patients with diverse etiologies of respiratory failure and diverse functional statuses. Alternatively, a non-paced 3-min sit-to-stand test (3-STST) could help to overcome this practical issue and be even more easily implemented in routine evaluation during a PR program. Indeed, although the test proposed by Aquilaniu et al. has been validated in its paced version [12], several teams, given the ease of implementation, actually perform a non-paced version in daily practice, with no data to support the relevance of this procedure.

As exercise training is intended to improve exercise tolerance and, as part of a PR program, improve HRQoL [15], we hypothesized that the clinical relevance of such a test (the non-paced 3-STST) could be assessed by investigating its relationship with HRQoL, as evaluated by the VQ11 questionnaire [16,17]. This study therefore aimed to (i) analyze the relationship between non-paced 3-STST performance and HRQoL, (ii) to identify determinants of non-paced 3-STST performance, and (iii) to analyze the evolution of non-paced 3-STST performance and HRQoL over the course of a community-based PR program. Also, given that the 6-MWT represents a reference in the evaluation of exercise capacity in patients with COPD [18], we decided to conduct a secondary analysis investigating the relationship between the non-paced 3-STST and 6-MWT performance.

## 2. Materials and Methods

### 2.1. Study Design and Participants

The study protocol was registered on Health Data Hub (registration number: F20230404135333; www.health-data-hub.fr, accessed on 4 April 2023), and a conformity declaration was provided to the Commission Nationale Informatique et Libertés (registration number: 11359531; www.cnil.fr, accessed on 15 March 2023).

This retrospective study was conducted in two community-based physiotherapy centers specialized in PR in the Bordeaux area, France. All patients referred to a participating center for ambulatory PR from 1 February 2019 to 1 February 2022 were screened for eligibility. Participants were included in the data analysis from the initial PR assessment if they met the following criteria: age >18 years old, referral to PR with a medical diagnosis of COPD, and completion of at least one initial PR assessment with no missing data regarding both the 3-STST and HRQoL questionnaire. The exclusion criteria included patients objecting to the use of their data and patients referred for a primary diagnosis other than COPD. The participants were included in the data analysis for evolution from initial to second PR assessments if they completed both first and second PR assessments with no missing data regarding the 3-STST and HRQoL questionnaire.

### 2.2. Data Collection

In accordance with usual care, we performed a standardized initial assessment at the beginning of PR, including: the collection of anthropometric data (age, height, weight, and gender); work situation; smoking status (history of tobacco consumption and current smoking situation); the use of oxygen (long-term oxygen therapy or ambulatory oxygen therapy); and a history of exacerbations during the previous year (defined according to the American Thoracic Society (ATS)/European Respiratory Society (ERS) criteria as episodes of increasing respiratory symptoms) [19]. Lung function was evaluated by spirometry (Spirobank II^®^ Basic, MIR, Langlade, France), and the following data were collected: forced expiratory volume in one second (FEV_1_), forced vital capacity (FVC), and FEV_1_/FVC ratio. The data were expressed in percentage of theoretical values (%pred) according to the ATS/ERS standards [20]. Exertional dyspnea was assessed with the modified Medical Research Council scale (mMRC) [21], and HRQoL was assessed with the VQ11 questionnaire according to national guidelines [16,17,22]. The VQ11 questionnaire is composed of 11 items distributed across three components (functional, psychological, and social), yielding a total score ranging from 11 to 55, with a total score > 21 indicating impaired HRQoL. The functional evaluation included the 6-MWT [9], during which dyspnea (modified Borg scale) and pulsed oxygen saturation (SpO_2_; Spirodoc Oxy^®^, MIR, Langlade, France) were collected at rest and at the end of the test. The total walked distance was recorded in meters and in percentage of theoretical values [23,24]. The BODE index was calculated from the body mass index (BMI), FEV_1_, dyspnoea (mMRC), and distance walked on the 6-MWT [18].

In line with usual care in the context of a community-based intervention, the PR program included exercise training, therapeutic education, and support for smoking cessation depending on the patient’s needs [4]. The exercise training sessions were performed two or three times a week and included 30 to 45 min of interval or constant-load endurance training (on a cyclo-ergometer or treadmill), as well as muscular strengthening of the limbs at 60 to 80% of maximal resistance. The treatment modalities were standardized between the two inclusion centres. A second PR assessment was carried out after 20 to 30 sessions, corresponding to a 6–8 weeks PR program or in between if a medical consultation was scheduled.

### 2.3. The Non-Paced 3-STST

The non-paced 3-STST was routinely implemented as part of the standard PR assessment. It consisted of standing up and down from a chair (height: 46 cm) without armrests as many times as possible for 3 min. The participants were given the following instructions: “*This test consists of sitting up and down from this chair as many times as possible for 3 min. Three minutes is a long time. If you feel the need to take a break you can, but I’ll keep the clock running. You may resume as soon as you wish. If you don’t feel you can finish the test, you are also allowed to stop. During the test I will not encourage you, but simply give you the elapsed time at 1 min, 2 min, and 2 min 30 s. To stand up and sit down, I ask you to do so without the help of your arms (you can leave your arms on your hips or cross them over your chest), to stand up completely (with your legs straight and your back straight), and to sit down completely. When you are ready, let me know, I will start the timer the first time you stand up*”. The participants started the test sitting down, and the total number of chair lifts was recorded as the primary outcome. In a non-systematic and unstructured process, the number of repetitions during the first minute of the 3-STST was also recorded.

### 2.4. Statistical Analysis

The normality of data distribution was tested using the Shapiro–Wilk test and by visual inspection of the Q-Q plots. Continuous variables are presented as the mean and standard deviation (SD) and categorical variables are presented as number and percentage (*n* [%]).

The association between the non-paced 3-STST performance (no. repetitions) and VQ11 total score (and its components considered separately) was tested by linear regression. The results are expressed as estimates and R coefficients with 95% confidence intervals (95%CI). The same approach was used to compare the non-paced 3-STST performance and the 6-MWT distance.

To identify determinants of 3-STST performance, we built a generalized linear model including the number of repetitions performed on the 3-STST as the dependent variable. All variables available at initial PR assessment listed in Table 1 and Table 2 were tested one by one and selected for the model if *p* < 0.1 and if the rate of missing data was <20%. Several indicators of dyspnea were available in the collected data. Therefore, consistently with the BODE index, we chose to retain in our model the dichotomisation of the mMRC (between values > and ≤1).

In the final model, the following assumptions were verified: linearity, absence of collinearity in the predictors, homoscedasticity, normality of residuals, absence of influential data points, and independence.

Finally, the comparison of variables between the initial and second PR assessments was performed for continuous variables using paired *t*-tests and Wilcoxon signed rank tests depending on the data distribution and McNemar tests for dichotomous data. The results are expressed as absolute values as well as the mean difference and 95%CI. The associations between the evolution from the initial to second PR assessments of VQ11 total score and 3-STST performance, as well as between the 6-MWT distance and the 3-STST performance, were tested by linear regression.

All statistical analyses were performed using JAMOVI software version 2.3.21 (www.jamovi.org; Gamjl package). *p*-values < 0.05 were considered statistically significant.

## 3. Results

### 3.1. Population Characteristics at Initial PR Assessment

**Figure 1:**
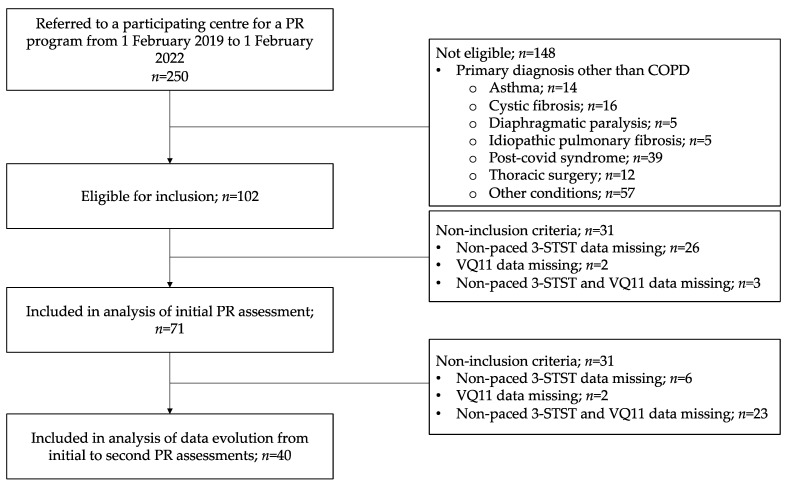
Study flow chart. Non-paced 3-STST, non-paced three-minute sit-to-stand test; COPD, chronic obstructive pulmonary disease; PR, pulmonary rehabilitation.

The flow-chart of included patients is presented in Figure 1. From 1 February 2019 to 1 February 2022, 250 patients were referred to a participating center for a PR program. Among these patients, 102 had a diagnosis of COPD, of whom 73 (72%) actually completed a 3-STST. Seventy-one patients were included in the analysis of data from the initial PR assessment (complete data for both 3-STST and VQ11).

The participants’ characteristics at the initial PR assessment are presented in Table 1 and Table 2. Most participants were retired (58%), with a mean age of 69 ± 10 years, and 39 (55%) were female. Approximately 36 (51%) participants had a FEV_1_ < 50%pred (GOLD spirometric stages III-IV), and 53 (75%) experienced dyspnea with mMRC > 1. Despite this, their functional status was moderately altered, with a mean distance walked on the 6-MWT of 412 ± 91 m, corresponding to 84 ± 19% of the theoretical values. Nevertheless, HRQoL, as assessed through the VQ11 questionnaire, was impaired in 58 (82%) participants.

The mean number of repetitions performed on the 3-STST at the initial PR assessment was 43 ± 15 (Table 2). We assessed the number of repetitions performed during the first minute of the 3-STST in only 40 (56%) participants, which was on average 19 ± 6 repetitions, ranging from 6 to 32.

### 3.2. Relationship between Non-Paced 3-STST Performance and Other Collected Variables

**Figure 2:**
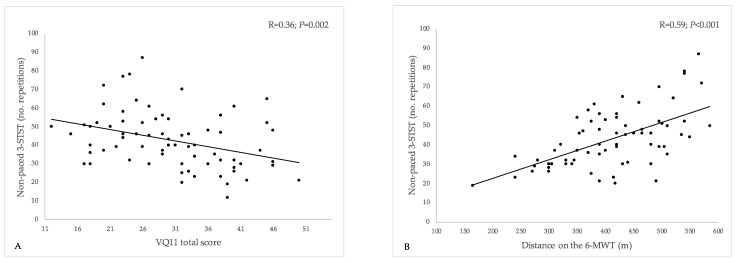
Relationship between the non-paced 3-min sit-to-stand test, health-related quality-of-life (VQ11 questionnaire), and 6-min walking test distance. 3-STST, non-paced three-minute sit-to-stand test; 6-MWT, 6-min walking test. (**A**) Relationship between 3-STST and VQ11 total score; (**B**) relationship between 3-STST and 6-MWT distance. A lower VQ11 score indicates better HRQoL.

Performance on the non-paced 3-STST was significantly and negatively correlated with the VQ11 total score (estimate [95%CI]: −0.61 [−1.00; −0.23]; R = 0.36 [0.13; 0.54]; *p* = 0.002) (Figure 2A). In other words, 3-STST performance was lower in patients with the most impaired HRQoL. This association was evidenced for all the three sub-components of the VQ11, namely functional (−2.29 [−3.44; −1.13]; R = 0.43 [0.21; 0.60]; *p* < 0.001), psychological (−1.49 [−2.61; −0.36]; R = 0.30 [0.07; 0.50]; *p* = 0.011), and social components (−1.01 [−1.92; −0.10]; R = 0.26 [0.02; 0.46]; *p* = 0.031).

The number of repetitions performed on the non-paced 3-STST was also significantly and positively correlated with the distance walked on the 6-MWT (0.10 [0.06; 0.13]; R = 0.59 [0.40; 0.72]; *p* < 0.001) (Figure 2B).

During the univariate analyses, the following variables were also associated with greater 3-STST performance: younger age (−0.53 [−0.90; −0.16]; R = 0.33 [0.10; 0.52]; *p* = 0.005), female gender (8.22 [1.15; 15.3]; R = 0.27 [0.04; 0.47]; *p* = 0.023), active smoking status (9.31 [1.33; 17.30]; R = 0.30 [0.04; 0.51]; *p* = 0.023), higher FEV_1_ (0.34 [0.15; 0.52]; R = 0.41 [0.19; 0.59]; *p* < 0.001), and exertional dyspnea with mMRC ≤ 1 (19.40 [10.30; 28.50]; R = 0.48 [0.26; 0.65] *p* < 0.001). The number of repetitions performed during the first minute of the 3-STST was significantly associated with the total number of repetitions performed at the end of the test (R = 0.82 [0.66; 0.91]; *p* < 0.001). It was also significantly associated with the 6-min walking test distance (0.03 [0.20; 0.88]; R = 0.54 [0.21; 0.76]; *p* = 0.003) but with no other BODE variables.

### 3.3. Determinants of the 3-STST Performance

During the multivariate analysis, younger age, exertional dyspnea with mMRC ≤ 1, and lower VQ11 total score (i.e., better HRQoL) remained significantly associated with better 3-STST performance (Table 3).

### 3.4. Evolution from Initial to Second PR Assessment

From the initial cohort, 40 (56%) patients were included in this secondary analysis (available data for both 3-STST and VQ11), with a mean duration between initial and second PR assessments of 29 ± 11 rehabilitation sessions. The evolution of the variables collected from the first to second PR assessment is presented in Table 2.

During PR, the number of repetitions performed on the 3-STST increased from 43 ± 15 to 48 ± 19 (*p* = 0.005). The participants also significantly improved their functional capacities, as evaluated by the distance walked on the 6-MWT (from 412 ± 91 to 456 ± 84 m [*p* = 0.002]), and their HRQoL (from 30 ± 9 to 27 ± 10 [*p* = 0.036]), as evaluated by the VQ11 questionnaire.

**Figure 3:**
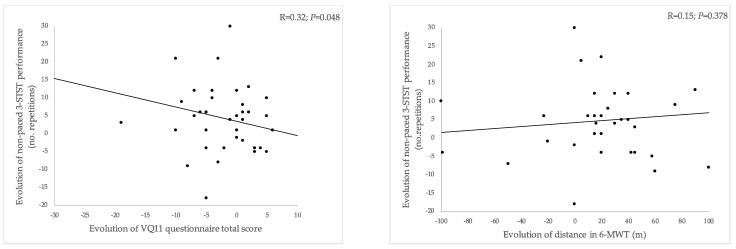
Relationship between changes in 3-STST performance from the first to second assessment compared to changes in VQ11 total score (**A**) and changes in 6-MWT distance (**B**). 3-STST, non-paced three-minute sit-to-stand test; 6-MWT, 6-min walking test. A reduced VQ11 score indicates improved HRQoL.

As shown in Figure 3, from the initial to second PR assessment, the improvement in 3-STST performance was significantly associated with the improvement in HRQoL (i.e., reduction in the VQ11 total score: *R* = 0.32 [0.00; 0.57], *p* = 0.048) (Figure 3A) but not with the evolution of the 6-MWT distance (*R* = 0.15 [−0.18; 0.45], *p* = 0.378) (Figure 3B).

## 4. Discussion

In this study, in which we processed data collected in a real-life setting, we hypothesized that the non-paced 3-STST, as part of a standard evaluation of a community-based PR program, could be considered clinically relevant if associated with HRQoL. Our results support this assumption; the performance on the 3-STST was associated with all the components of the VQ11 questionnaire. It is noteworthy that the association between 3-STST performance and VQ11 total score held true after adjustments for other relevant covariates, and changes in these two parameters over the course of PR were significantly correlated. Non-paced 3-STST performance was also associated with most of the markers of clinical severity constituting the BODE index, suggesting that this test provides relevant and valuable information in the evaluation of patients with COPD referred to a community-based PR program. The confidence intervals for these associations were nevertheless quite large, and further sufficiently powered prospective studies are needed to confirm these results. Finally, the non-paced 3-STST was actually performed in nearly three fourths of the patients referred to a participating center during the inclusion period, supporting its feasibility in routine practice.

Of note, after adjustment for other covariates, the severity of airflow obstruction as assessed by the FEV_1_ was not associated with the number of repetitions performed on the 3-STST. It also did not correlate with the spontaneous pace adopted by the patients during the first minute of the test. In a study aimed at identifying the minimal clinically important difference (MCID) of the paced 3-STST as initially described [12], Lévesque et al. suggested that the pace imposed on the first minute of the paced 3-STST could be chosen based on the presence or absence of an FEV_1_ < 1000 mL (12 or 20 repetitions, respectively) [14]. Our results do not support this assumption. Moreover, in the princeps study of Aguilaniu et al., the number of repetitions performed on the paced 3-STST with a pace of 12/min imposed on the first minute was 49 ± 10, compared to 55 ± 11 when imposing a pace of 20/min [12]. The observed difference was above the suggested MCID of this test (five repetitions), which questions the relevance of imposing the pace of the first minute. In addition, our data suggest that the pace spontaneously adopted by the patients during the first minute is strongly correlated with the final performance, which reinforces our hypothesis that, in this population of patients referred to a community-based PR program, imposing the first minute pace is questionable. Our data also indicate that this non-supervised pace can be quite variable (range 6–32), and starting the 3-min STST too quickly or too slowly can obviously affect the final performance, which is the reason why Aguilaniu et al. had chosen to impose a pace. To some extent, this variability might be addressed by conducting a preliminary test to reduce the learning effect. We did not address this issue in the present study, which should be considered as a limitation and taken into account when conducting future studies on this topic. Also, prospective comparison of the paced and non-paced 3-STST could help to clarify the respective clinical relevance of these two versions of the test.

Another important consideration supporting the use of a non-paced version of the test is the ability to compare results with those obtained in other patient populations. For instance, we previously reported data from a cohort of patients referred to PR for COVID-19-related persistent dyspnea, in which patients without prolonged functional sequelae had a mean 3-STST performance of only 47 ± 21 repetitions [25]. Had we been able to compare these data with our current study population, this would obviously have provided material for discussion.

Our study has several limitations. First of all, our data represent only a first step in the process of validating this non-paced 3-STST test for clinical practice, which should be further complemented by assessing the test’s reproducibility and accuracy compared to a reference [26]. Selecting the appropriate comparative reference test is an important step, for which our data provide methodological guidance. Indeed, if the reference standard to assess the functional capacities of patients with COPD patient is undoubtedly the 6-MWT [27], our data show that after adjustment for other co-variates, the distance walked on the 6-MWT poorly explained performance on the non-paced 3-STST. This suggests that, although associated with the 6-MWT distance, the non-paced 3-STST provides information that is complementary to the walking test rather than being a surrogate. Also, the respective changes in distance covered in the walking test and performance in the non-paced 3-STST were not significantly correlated. Some confounding factors may explain this observation, in particular exertional dyspnea, which appears to be a major determinant of performance on the non-paced 3-STST. Unfortunately, we did not monitor relevant indicators such as SpO_2_, heart rate, or dyspnea during the 3-STST, which in view of our results would be relevant in a next step [28].

Finally, further studies would be helpful in better defining what the non-paced 3-STST actually measures. Indeed, performance in this test may be an indicator of not only lower limb strength, postural control, exercise capacity but also dyspnea and quality of life as suggested by our results. Also, choosing the most appropriate sit-to-stand test may be challenging, with several versions being described, ranging from short versions (5–10 repetitions) and middle duration (30 s–1 min) to longer 3-min versions [12,29]. The 1-min STST, which is the most widely used and validated sit-to-stand test in pulmonary rehabilitation [29], is primarily an indicator of muscular performance and is much less hemodynamically stressful than field tests addressing exercise capacity such as the 6-MWT or the paced 3-STST [10,26,27]. Our assumption is that the limiting factors for the non-paced 3-STST are likely to be at the boundary between ventilatory and muscular limitation. In our population of patients with respiratory insufficiency, this dual limitation constitutes the main rationale for choosing this test rather than shorter ones. Consistent with our observations, future studies aimed at validating the non-paced 3-STST should thus not restrict their analysis to physical performance indicators and should also consider exertional dyspnea, which appears to substantially contribute to explaining performance in this test.

## 5. Conclusions

This study describes the non-paced 3-STST, whose performance appears to be associated with HRQoL and other markers of clinical severity in individuals with COPD referred to a community-based PR program. The data presented support the feasibility of this test in daily practice, and further studies are required to determine the clinical validity of this test.

## Figures and Tables

**Table 1:** Participants’ baseline characteristics.

	First PR Assessment *n* = 71	Missing Data *n* (%)
General characteristics		
Age (years old)	69 ± 10	0 (0)
Gender (female); *n* (%)	39 (55)	0 (0)
BMI (kg·m^−2^)	25 ± 6	0 (0)
Smoking history (yes); *n* (%)	62 (87)	0 (0)
Tobacco consumption (PY)	46 ± 23	28 (39)
Active smoking status; *n* (%)	22 (37)	12 (17)
Oxygen therapy; *n* (%)	–	1 (1)
LTOT	11 (16)	–
Ambulatory OT	13 (19)	–
Exacerbations per year (no.)	1 ± 1	27 (38)
Respiratory evaluation		
FEV_1_ (%pred)	49 ± 19	2 (3)
FVC (%pred)	73 ± 21	9 (13)
FEV_1_/FVC (%)	53 ± 13	9 (13)
mMRC	2 ± 1	7 (10)
mMRC > 1; *n* (%)	53 (75)	7 (10)
BODE index	4 ± 2	11 (15)

Legend: BMI, body mass index; FEV_1_, forced expiratory volume in one second; FVC, functional vital capacity; LTOT, long-term oxygen therapy; mMRC, modified medical research council scale; OT, oxygen therapy; PR, pulmonary rehabilitation; PY, pack-year.

**Table 2:** Evolution from first to second PR assessments.

Variables	First PR Assessment *n* = 71	Missing Data *n* (%)	Second PR Assessment *n* = 40	Missing Data *n* (%)	Mean (95%CI) Difference	*p*-Value
Functional evaluation						
6-MWT	–	–	–	–	–	–
Distance (m)	412 ± 91	3 (4)	456 ± 84	3 (8)	30 (12; 47)	0.002 *
Distance (%pred)	84 ± 19	3 (4)	91 ± 15	5 (13)	6 (2; 10)	0.002 *
Resting SpO_2_ (%)	95 ± 2	5 (7)	95 ± 2	5 (13)	−0 (−2; 1)	0.719 ^†^
Minimum SpO_2_ (%)	84 ± 6	5 (7)	86 ± 5	5 (13)	1 (−1; 3)	0.174
Resting HR (/min)	91 ± 16	5 (7)	89 ± 17	5 (13)	−1 (−4; 2)	0.394
Maximum HR (/min)	129 ± 23	5 (7)	131 ± 28	6 (15)	3 (−6; 12)	0.519
Resting dyspnea (mBorg)	1 ± 2	4 (6)	1 ± 2	5 (13)	0 (−1; 2)	0.727 ^†^
End-test dyspnea (mBorg)	5 ± 2	4 (6)	5 ± 2	5 (13)	0 (−1; 1)	0.636 ^†^
Resting leg fatigue (mBorg)	0.5 ± 1	5 (7)	0.6 ± 2	5 (13)	1 (−2; 4)	0.509 ^†^
End-test leg fatigue (mBorg)	3 ± 3	5 (7)	3 ± 3	5 (13)	−2 (−4; 1)	0.243 ^†^
Non-paced 3-STST (no. repetitions)	43 ± 15	0 (0)	48 ± 19	0 (0)	4 (1; 7)	0.005 *
First minute of the non-paced 3-STST (no. rep)	19 ± 6	40 (56)	23 ± 7	24 (60)	3 (0; 6)	0.069
Health related quality-of-life						
VQ-11 questionnaire	–	0 (0)	–	0 (0)	–	–
Functional dimension	10 ± 3	–	9 ± 4	–	−0 (−1; 1)	0.554
Psychological dimension	11 ± 3	–	10 ± 3	–	−1 (−2; −0)	0.047 *
Social dimension	9 ± 4	–	8 ± 4	–	−1 (−3; 1)	0.278 ^†^
Total score	30 ± 9	–	27 ± 10	–	−3 (−5; −0)	0.036 *
VQ-11 > 21; *n* (%)	58 (82)	–	25 (63)	–	–	0.058

Legend: 3-STST, three-minute sit-to-stand test; 6-MWT, six-minute walking test; HR, heart rate; mBorg, modified Borg scale; SpO_2_, pulsed oxygen saturation. * p-values < 0.05. ^†^ Wilcoxon signed rank test.

**Table 3:** Multivariate model identifying the determinants of 3-STST performance in the study population at the initial PR assessment.

	3-STST (R^2^ [95%CI] = 0.58 [0.45; 0.71])	
Variable [Reference]	Estimate (95%CI)	*p*-Value
Age (year old)	−0.51 (−0.95; −0.07)	0.025 *
Gender [female]	−2.07 (−9.45; 5.32)	0.575
Active smoking [yes]	0.56 (−6.61; 7.74)	0.875
FEV_1_ (%pred)	0.19 (−0.01; 0.40)	0.068
mMRC ≤ 1 [yes]	9.55 (1.14; 17.96)	0.027 *
6-MWT distance (m)	0.03 (−0.02; 0.08)	0.210
VQ11 total score	−0.53 (−0.94; −0.13)	0.010 *

Legend: 3-STST, three-minute sit-to-stand test; 6-MWT, six-minute walking test; 95%CI, 95% confidence interval; FEV1, forced expiratory volume in one second; mMRC, modified Medical Research Council scale; OT, oxygen therapy. * *p*-values < 0.05.

## Data Availability

After deidentification, individual participant data (including data dictionaries) that underlie the results reported in this article (text, tables, and figures), including the study protocol, will be made available to researchers who provide a methodologically sound proposal to achieve aims in the approved proposal, beginning immediately following article publication and ending 5 years following article publication. Proposals should be directed to the corresponding author. To gain access, data requestors will need to sign a data access agreement.
